# Dust Mite Serodominance Profiles in Lugo

**DOI:** 10.1002/iid3.70254

**Published:** 2025-10-15

**Authors:** Francisco Carballada, Luis Alfredo González, Ramón Núñez, Raquel Lopez, Joaquín Martín, Nicola Giangrande, Antonio García‐Dumpierrez, Javier Alcover, David Rodríguez, Ricardo Palacios

**Affiliations:** ^1^ Sección de Alergia del Hospital Universitario Lucus Augusti de Lugo España; ^2^ Hospital da Costa Burela Lugo España; ^3^ Hospital Universitario Dr. Negrín Gran Canaria España; ^4^ Laboratorios DIATER Leganés Madrid España

**Keywords:** allergy, HDM, IgE, serodominance, seroprevalence

## Abstract

**Introduction:**

House dust mite allergy affects 65–130 million people worldwide. Untreated patients could develop severe allergic diseases such as atopic dermatitis and asthma.

**Methods:**

We consecutively recruited 50 patients with a clinical diagnosis of allergic rhinitis/rhino conjunctivitis. Mite Skin Prick Tests were performed. Mite specific IgE were tested.

**Results:**

Ninety‐six per cent of the patients had a positive prick test for Dpt, Dfar 94%, Ldt 86%, Tput 82%, and Blot 82%. Der p1 was recognized by 70%, Der p2 84%, Der p23 72%. Regarding serodominances, Der p2 was the highest one (30.2 kU/L). Forty‐three patients presented sensitization to 3 *Dermatophagoides pteronyssinus* molecules, been 1 + 2 + 23 the main combination. Der p 1, 2, and 23 prevalence were similar in patients with intermittent or persistent allergic rhinitis. All patients with asthma recognized to Der p2 and more than 80% Der p1 and 23. In the group of patients without asthma, sensitivity to Der p23 is considerably reduced.

**Conclusion:**

Ninety per cent of patients recognize Der p1, 2, and 23, alone or in any of their combinations. The high prevalence of sensitization to Der p23 (72%) stands out, that all asthmatic patients are sensitive to Der p2 and that sensitization to Der p23 in non‐asthmatic patients drops to 50%.

## Graphical abstract

We consecutively recruited 50 patients with a clinical diagnosis of allergic rhinitis/rhino conjunctivitis. Der p1 was recognized by 70%, Der p2 84%, Der p23 72%.

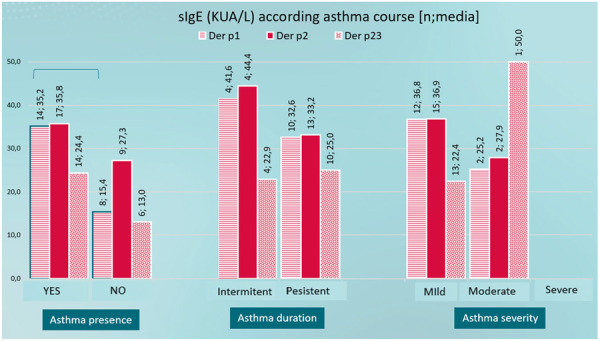

House dust mite (HDM) allergy affects 65–130 million people worldwide, near to 1.5% of the world population. No treated patients could develop severe allergic diseases such as atopic dermatitis and asthma [1].

Humidity is an essential factor for the development of mites. Specifically, domestic mites have adapted to the living conditions of humans. In the case of Galicia, there are different macro bioclimates and bioclimates [2]: temperate (sub‐Mediterranean), hyper oceanic; oceanic temperate; temperate (sub‐Mediterranean) oceanic and pluvial‐seasonal oceanic Mediterranean, associated with high levels of sensitization, predominantly in coastal areas. Additionally, the rainfall in the territory in general aggravates the problem. In previous works, HDM serodominance profiles have been evaluated in Coruña [3], a coastal area with a temperate hyper oceanic climate. In this study, a similar study has been carried out in Lugo, an inland area with a temperate oceanic climate, as a preliminary step to a larger scale study in the entire province of Galicia.

More than 90% of allergies to HDM around the world are caused by *Dermatophagoides pteronyssinus* (Dpt) and *Dermatophagoides farinae* (Dfar) [4]. Dpt has 34 and Dfar 37 allergens described according to the Allergen Nomenclature Subcommittee of the World Health Organization and the International Union of Immune Societies. There are wide variations in the prevalence rates of the main immunodominant allergens [5].

Exposure to allergens depends on multiple factors such as climate and mite microhabitats within the domestic environment. In Galicia Dpt has a significant clinical importance [6]. Thirty‐two species were identified. The main specie was Dpt, which was present in 97.6% of dust samples, with 12–13 μg per gram of dust of Der p 1 and 1.1–1.5 μg per gram of Der f 1. Humidity in the bedrooms and the age of the mattress increased the number of mites. *Cheyletus* species, *Euroglyphus maynei*, *Lepidoglyphus destructor* (Ldt), and *Chortoglyphus arcuatus* or, much less, *Blomia tropicalis* (Blot) were also found [7, 8].

Allergy Explorer2 (Macro Array Diagnostics, Wien, Austria) a multiparametric assay containing an extended panel of HDM allergens (including Der f 1, Der p 1, Derf 1, Der f 2, Der p 2, Lep d 2, Der p 5, Blo t 5, Der p 7, Der p 10, Blo t 10, Der p 11, Der p 20, Der p 21, Blo t 21, Der p 23) was used for HDM molecular diagnosis. In the present study, we also used immunoblotting assays.

## 1 Materials and Methods

### 1.1 Patients' Selection and Sampling

To establish the molecular serodominance profile in patients with dust mite allergy symptoms in Lugo, we consecutively recruited 50 patients with a clinical diagnosis of allergic rhinitis/rhino conjunctivitis and/or allergic asthma according to the ARIA and GINA guidelines [9, 10] from the allergy department of Hospital Universitario *Lucus Augusti* Lugo in Lugo, Spain. All patients had to fulfil the following clinical criteria: (a) confirmed diagnosis of rhinitis, rhino conjunctivitis, and or asthma [9, 10], (b) positive skin prick tests (SPTs) for Dpt (a prick test is considered positive when a papule with a diameter greater than 3 mm is produced) and/or Dpt specific IgE (sIgE) ≥ 0.35 kU_A_/L, (c) reside in our health area for at least 3 years before inclusion in the study, and (d) aggravation of symptoms by contact with indoor dust. No patients underwent to previous HDM‐specific immunotherapy.

Serum samples were obtained by blood clotting and centrifugation at 3000 rpm for 10 min at 4°C. Then, they were stored at −80°C until use before in vitro analysis. The local clinical research ethics committees approved the project (CEIC Complexo Hospitalario Universitario A Coruña) on March 25, 2021 (code number 2021/169). Patients/caregivers received all necessary information, and they signed a form of written informed consent to participate.

### 1.2 Intraepidermal Test

SPTs were performed according to European standards with standardized extracts of Dpt, Dfar, Ldt, *Tyrophagus putrescentiae* (Tput), and Blot (DIATER, Madrid, Spain). Histamine (10 mg/mL) and saline were used as positive and negative controls. Following daily clinical practice, antihistamines were withdrawn a week before the SPT. The wheel diameters were measured after 20 min, and diameters ≥ 3 mm were considered positive.

### 1.3 Mite Allergen Extracts

Proteins from mite bodies (Dpt and Ldt) were extracted in phosphate‐buffered saline, 0.01 M, pH 7.4, for 2 h at 5°C ± 3°C. Both protein solutions were clarified by filtration and centrifugation against highly purified water (European Pharmacopoeia specification), sterile filtered, frozen, and lyophilized.

### 1.4 SDS‐PAGE/IgE Western Blot

Proteins from Dpt and Ldt extracts were analysed using sodium dodecyl sulfate‐polyacrylamide gel electrophoresis according to Laemmli [11] in 15% polyacrylamide gels under reducing conditions. Proteins were visualized using Coomassie Brilliant Blue R‐250 staining and transferred to polyvinylidene difluoride membranes (Trans‐Blot Turbo; Bio‐Rad, Hercules, CA, USA). The binding of IgE antibodies to allergens was analysed using Western blot with individual patients' sera and antihuman IgE peroxidase conjugate (Southern Biotech, Birmingham, AL, USA). Chemiluminescence detection reagents (Western Lightning Plus‐ECL; Perkin Elmer, Madrid, Spain) were added following the manufacturer's instructions. IgE binding bands were identified using the Bio‐Rad Diversity database program.

### 1.5 In Vitro Assays

sIgE for Der f 1, Der p 1, Derf 1, Der f 2, Der p 2, Lep d 2, Der p 5, Blo t 5, Der p 7, Der p 10, Blo t 10, Der p 11, Der p 20, Der p 21, Blo t 21, Der p 23 were tested in sera of all patients using the last version of the multiparametric assay Allergy Explorer2 (Macro Array Diagnostics, Wien, Austria). In this system, the allergens are spotted onto a nitrocellulose membrane in a cartridge chip, which is incubated with 0.5 mL of a 1:5 dilution of serum under agitation. After 2 h of incubation, the chip is extensively washed and a pre‐tittered dilution of antihuman IgE labelled with alkaline phosphatase is added and incubated for 30 min. Following further washing, the enzyme substrate is added, and after 8 min, the reaction is completed. The membrane is dried and the intensity of color reaction for each allergen is measured by a coupled charged device camera [12]. A dedicated software digitalizes the images and produces a report listing components and they score in kU_A_/L (range 0.3–50 kU/L). Values above 0.35 kU_A_/L were considered positive.

### 1.6 Statistical Analysis

The IgE sensitization to the 16 different HDM molecules was evaluated in terms of prevalence in all subjects. Measures were separately in subjects with age, rhinitis, and asthma (in both cases frequency and severity. To study whether the frequency of observations is significantly different between 2 or more groups in specific IgE prevalence *χ* ^2^ tests for categorical variables was measured. When samples were smaller than 5 Fisher's exact tests were conducted for this matter. Differences in IgE levels between groups were analysed with the Mann–Whitney *U* test. *χ* ^2^ tests and Fisher′s exact tests were also calculated for every house dust mite and its recombinant allergens when divided into positive or negative, using 0.35 kU_A_/L as a cut‐off point. A *p‐*value of less than 0.05 was considered significant. Statistical calculations were performed with GraphPad Software (San Diego, CA, USA) and IBM SPSS Statistics 25 (IBM, New York, NY, USA).

## 2 Results

### 2.1 Demographic Characteristics

Fifty patients met the inclusion and exclusion criteria, with an age average of 28.5 ± 14.8; 13 (26%) under eighteen years; 64% were female. All the patients present rhinitis (20% intermittent, 78% persistent), 34% (17) present asthma (8% intermittent, 26% persistent).

### 2.2 Skin Prick Tests

Forty‐eight patients (96%) had a positive prick test for Dpt, 94% (48 patients) a positive prick test for Dfar, 86% (43) a positive prick test for Ldt, 82% (41) a positive prick test for Tput, and 82% (41) a positive prick test for Blot.

### 2.3 Allergen Recognition

**Figure 1:**
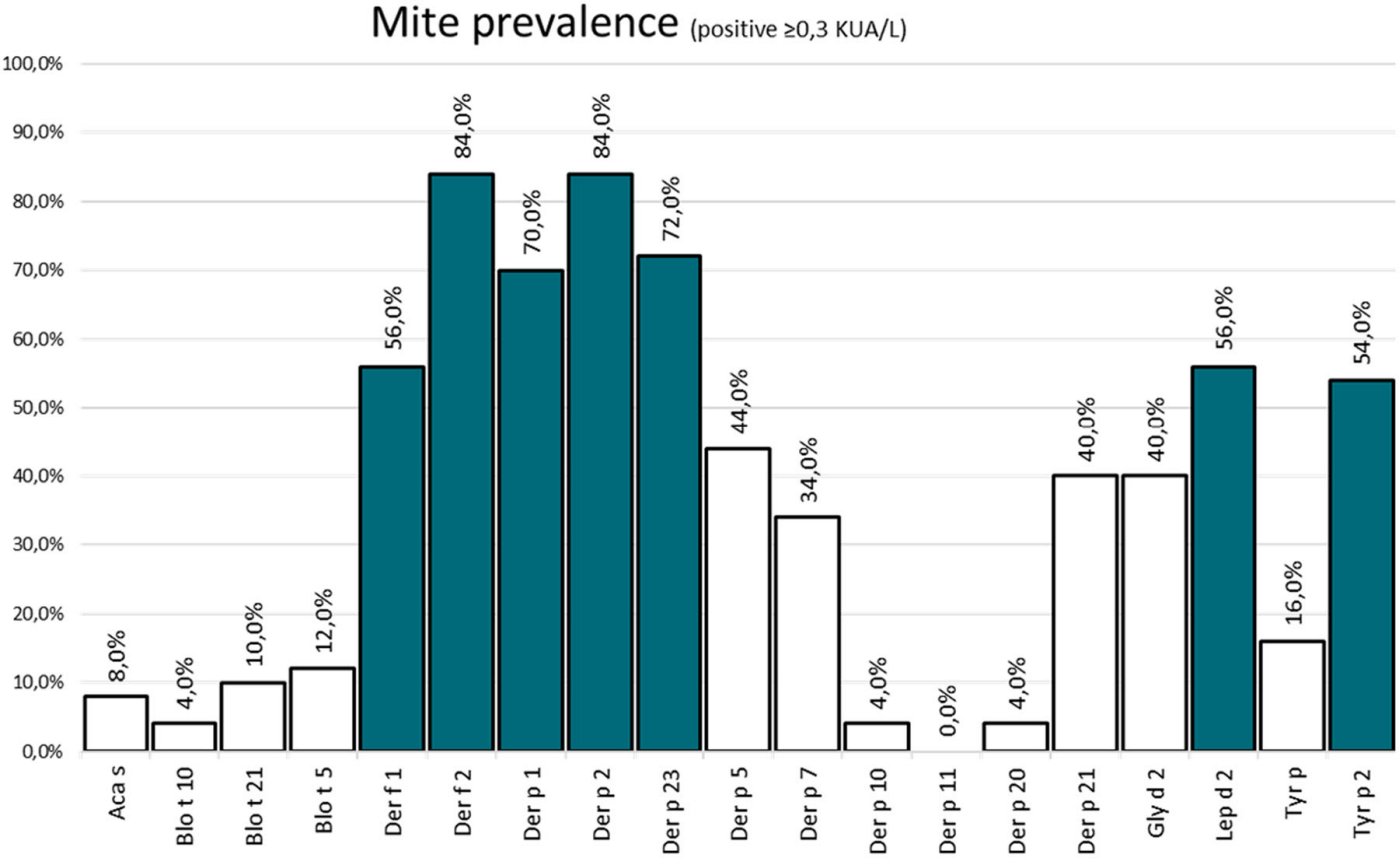
Mite molecule seroprevalence of the study population.

Mite molecule seroprevalence is shown in Figure 1. Der p 1 was recognized by 70% (35 patients) of the patients, while Der p 2 by 84% (42), Der p 23 by 72% (36), Der p 5 by 44% (22), Der p 7 by 34% (17), Der p 10 by 4% (2), Der p 11 by 0%, Der p 20 by 4% (2), Der p 21 by 40% (20). Regarding Ldt, Lep d 2 was recognized by 56% (28) of our patients, while in Blot, Blo t5 was recognized by 12% (6), Blo t 10 by 4% (2), and Blo t 21 by 10% (5 patients).

Among the patients with a positive prick test for Tput, total Tyr p was recognized by 16% (8), Tyr p 2 was recognized by 54% (27) of the patients.

Regarding serodominances (sIgE levels for each molecule). Der p 2 was the highest one (30.2 kU_A_/L), followed by Der p 20 (25.9), Der p 5 (23.2), Der p 1 (22.4) and Der p 23.

### 2.4 *D. pteronyssinus* Profiles

Fourty‐three per cent of the patients presented sensitization to 3 *D. pteronyssinus* molecules, with Der p 1 + Der p 2 + Der p 23 being the main combination. In second and third place are the patients who presented sensitization to 5 molecules (20%) and those who presented sensitization to 6 molecules (18%). There were only 4 monosensitized patientes (8%), 2 against Der p 2 (4%) and 2 against Der p 23 (4%).

Considering the profiles that include Der p 1, 2, and 23, alone or their combinations, more than half of the patients (60%) recognized the 3 allergens at the same time and 4 (8.0%) Der p 2; 2 (4.0%) Der p 23; 1 (2.0%) Der p 1; 4 (8.0%) Der p 1 + 2 and 4 (8.0%) Der p 2 + 23.

#### 2.4.1 Der p 1, 2, and 23 Prevalence by Age Groups [*n*; %]

**Figure 2:**
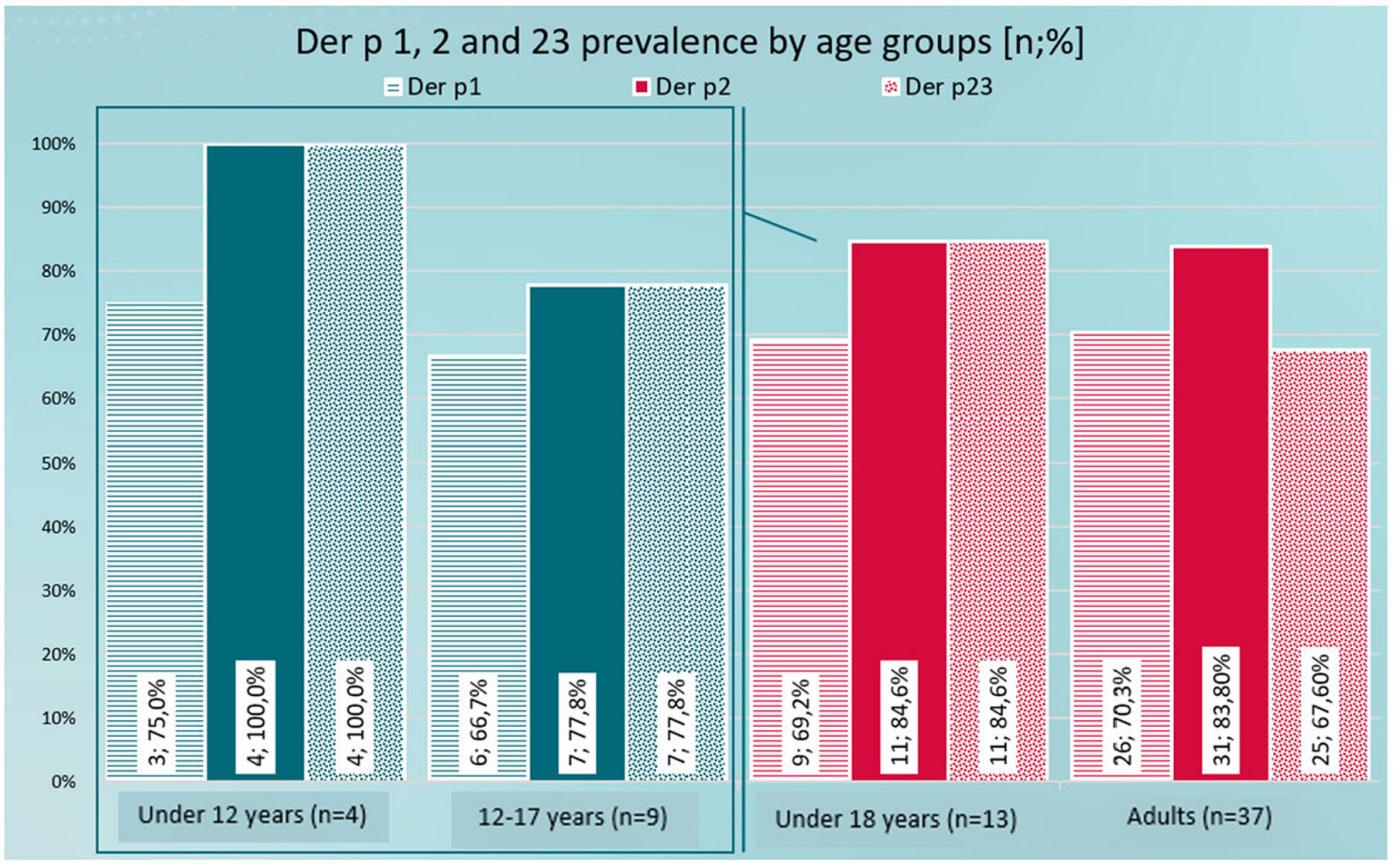
Der p 1, 2, and 23 prevalence by age groups.

If we analyse the prevalence of Der p 1, 2, and 23 in the different age groups (Figure 2), we observe that Der p 2 is the allergen to which the highest percentage of patients in any age group are sensitive. Underaged individuals are equally sensitized to Der p 2 and Der p 23, with Der p 1 being the allergen with a lower percentage of sensitive patients. However, in the adult group, sensitization to Der p 1 is higher than that of Der p 23. There are no statistically significant differences.

Regarding the combinations (different profiles) of Der p 1, 2, and 23 according to the age groups, although the percentage of patients sensitive to the combination Der p 1 + 2 + 23 is very similar in the adult and paediatric populations (61.5%; 59.5%), it should be noted that this percentage is much higher in those under 12 years of age (75%). More samples would be necessary to confirm whether this superiority is statistically significant. Also, in the Der p 2 + 23 combination, the percentage of sensitized people is much higher in children under 12 years of age. A larger sample would also be necessary for a better analysis. It should be noted that while sensitization to Der p 1 (only) appears in the group of patients between 12 and 17 years; The Der p 1 + 2 combination only does so in adult patients. There are no statistically significant differences.

### 2.5 Relation of Sensitization Profile to IgE With Clinic

All patients presented allergic rhinitis (AR). The main combination of AR is Persistent‐Mild or Persistent‐Moderate (39 patients). Der p2 is the allergen to which a higher percentage of patients are sensitive regardless of the duration of AR. The prevalence of Der p 1, 2, and 23 is very similar in the group of patients with intermittent AR and the group with persistent AR. There are no statistically significant differences.

Regarding duration, the main combination of AR is Persistent‐Mild or Persistent‐Moderate. In most allergen combinations, the prevalence is very similar in the group of patients with intermittent AR and in the group with persistent AR. However, the group of patients with intermittent AR has a higher percentage (although not significant) of patients without sensitivity to any of the allergens (Der p 1, 2, or 23) compared to the group of patients with persistent AR. Furthermore, the group of patients with intermittent AR does not present sensitivity to a single allergen.

**Figure 3:**
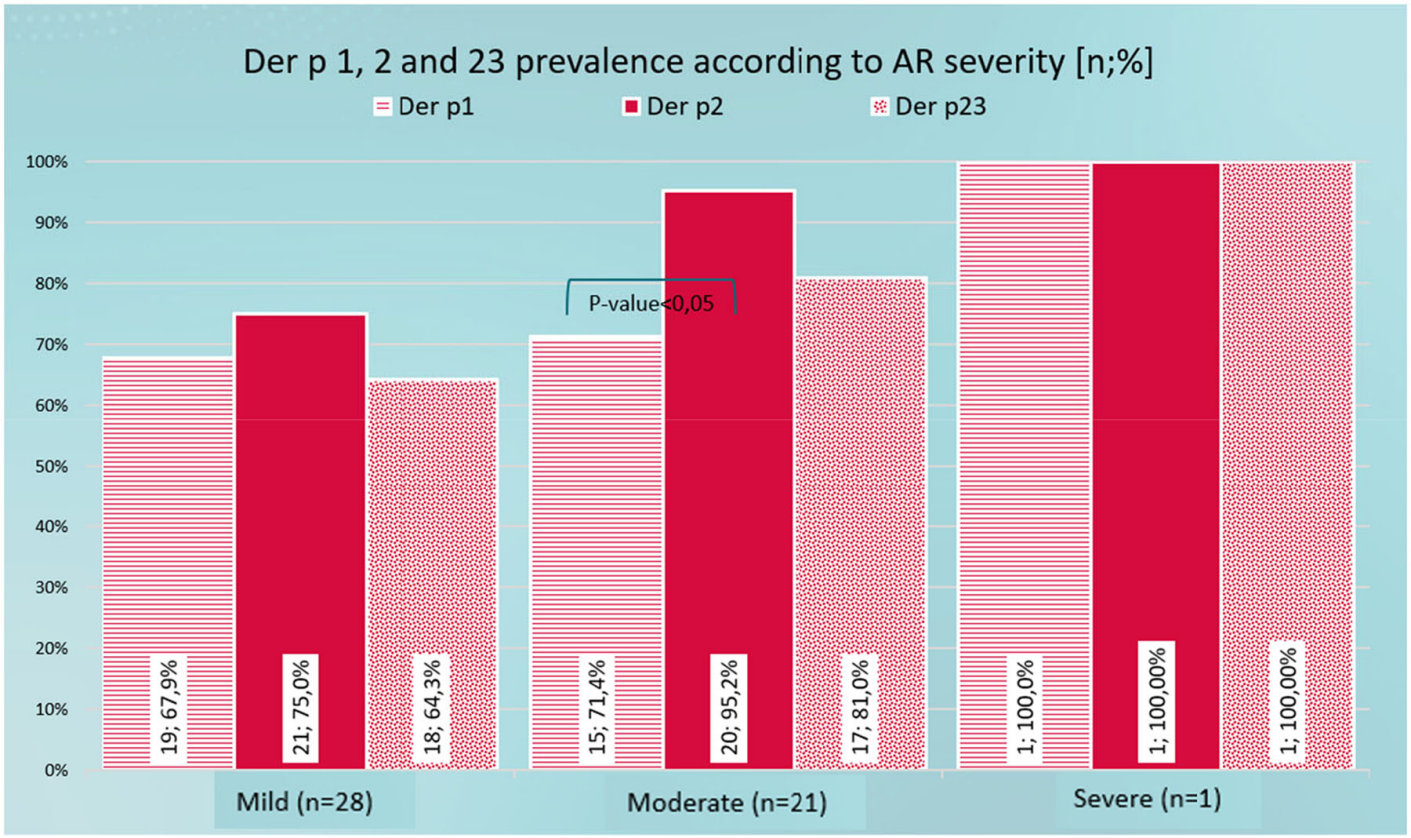
Der p 1, 2, and 23 seroprevalence according to AR severity.

The main combination of AR is Persistent‐Mild or Persistent‐Moderate. Der p 2 is the allergen to which a higher percentage of patients are sensitive regardless of the severity of AR. Furthermore, it is significantly higher than the prevalence of Der p 1 in patients with moderate AR severity. The prevalence of Der p 1 is very similar in patients with mild and moderate severity. However, the prevalence of Der p 2 and Der p 23 is higher in patients with moderate severity (Figure 3).

#### 2.5.1 Der p 1, 2, and 23 Prevalence According to Asthma Presence [*n*; %]

**Figure 4:**
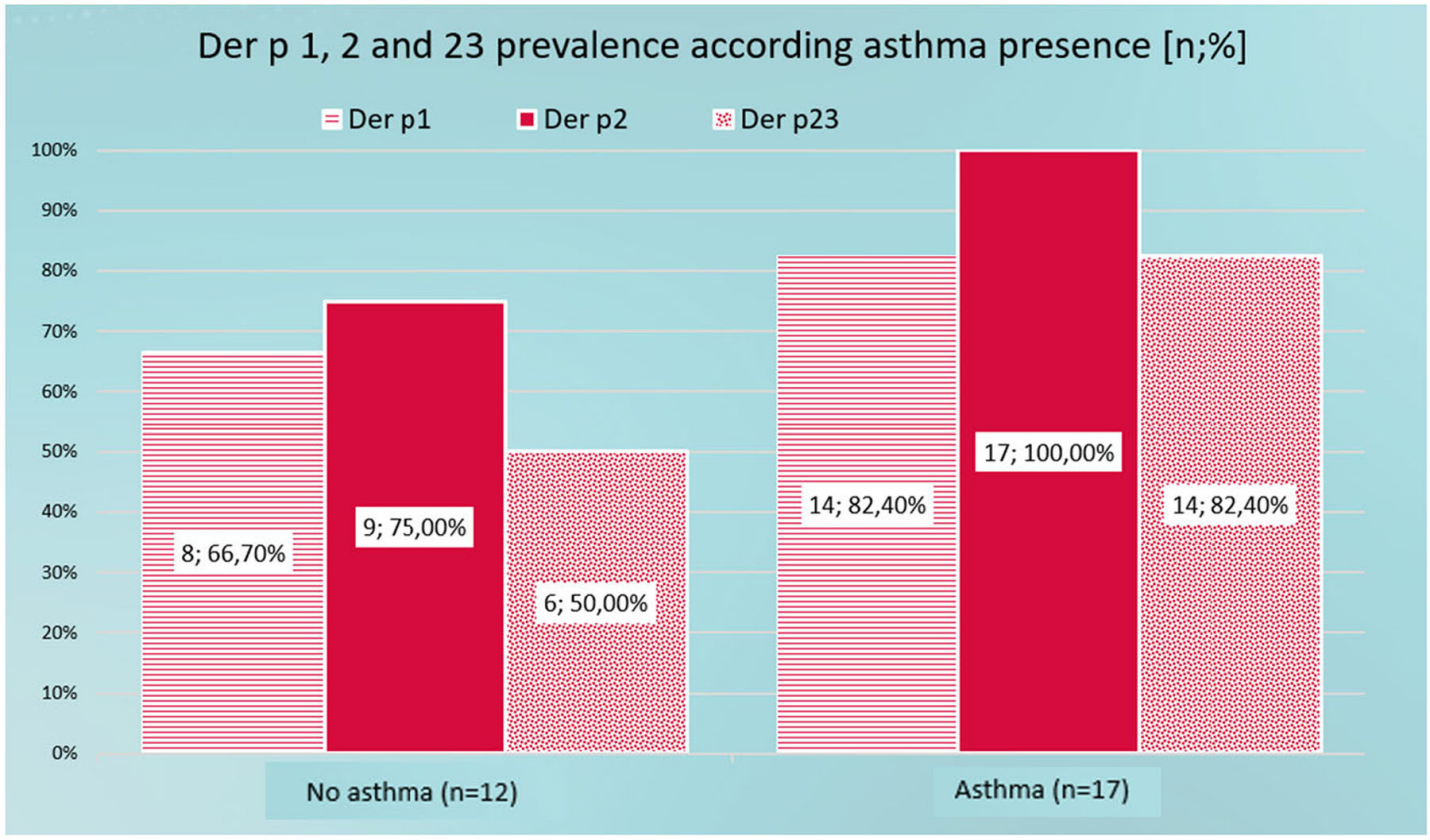
Der p 1, 2, and 23 seroprevalence according to asthma presence.

All patients with asthma presented sensitivity to Der p 2 and more than 80% presented sensitivity to Der p 1 and Der p 23. It should be noted that in the group of patients without asthma, sensitivity to Der p 23 is considerably reduced by up to 50%. This difference is close to statistical significance. A larger sample could present significant differences. There are no statistically significant differences (Figure 4).

In most allergen combinations the prevalence is very similar in the group of patients with asthma and without asthma. Although the prevalence of the Der p 1, 2, and 23 combination is higher in the group of patients with asthma, it is not statistically significant. However, the group of patients without asthma presents a higher and significant percentage of patients without sensitivity to any of the allergens (Der p 1, 2, or p23) compared to the group of patients with asthma. Furthermore, the group of patients without asthma only presents sensitization to the combinations and only one patient presents sensitivity to Der p 2 only.

#### 2.5.2 Der p 1, 2, and 23 Prevalence Related With Asthma Duration [*n*; %]

The main combination of asthma is Persistent‐Mild. Der p 2 is the allergen to which a higher percentage of patients are sensitive regardless of the duration of asthma. The prevalence of Der p 2 in the group of patients with persistent asthma is higher, 100% (and close to statistical significance) compared to the rest of Der p molecules in this group (Der p 1, 76.9% and Der p 23 76.9%).

#### 2.5.3 Prevalence of Combinations According to Duration of Asthma [*n*; %]

The main combination of asthma is Persistent‐Mild. All patients with intermittent asthma present sensitivity only to the combination Der p 1 + 2 + 23. The prevalence of the combination Der p 1 + 2 + 23 in the group of patients with persistent asthma is lower, 61.5% (but not significant) compared to the group with intermittent asthma, 100%.

#### 2.5.4 Prevalence Der p 1, 2, and 23 According to Asthma Severity [*n*; %]

The main combination of asthma is Persistent‐Mild. Der p 2 is the allergen to which a higher percentage of patients are sensitive regardless of the severity of asthma. Furthermore, it is higher (and close to statistical significance) than the prevalence of Der p 1 in patients with mild severity. A larger sample could present significant differences. The prevalence of Der p 1 is very similar in patients with mild and moderate severity. However, the prevalence of Der p 23 is higher in patients with mild severity (86%) compared to patients with moderate severity (50%).

#### 2.5.5 Prevalence of Combinations According to Severity of Asthma [*n*; %]

The main combination of asthma is Persistent‐Mild. The prevalence of the combination Der p 1 + 2 + 23 is higher, 23.3% (nonsignificant) in patients with mild asthma compared with moderate asthma, 50%.

**Figure 5:**
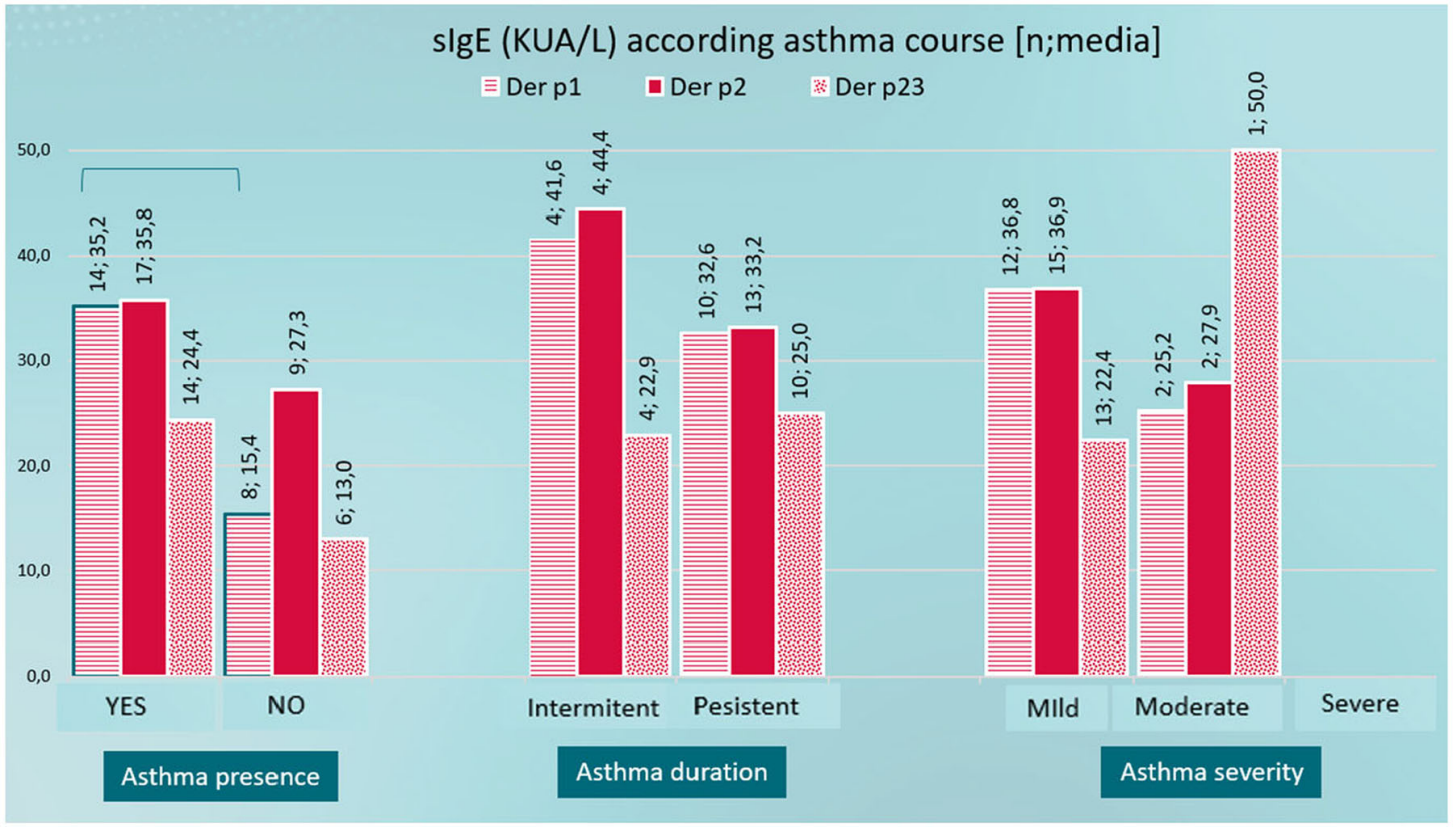
Der p 1, 2, and 23 serodominace according to asthma course.

Regarding Der p 1, 2, and 23 serodominance (sIgE KUA/L) according to asthma course, Der p 1 is statistically higher in patients with asthma and Der p 23 is higher in patients with moderate severity compared with mild severity (Figure 5).

To calculate the average sIgE (KUA/L), the data “> 50” has been considered as a value of 50 KUA/L. Allergens are NOT exclusive; That is, a patient will be counted in Der p 1 (if he is sensitive to the allergen) as well as in Der p 2 if he is also sensitive to this other allergen.

### 2.6 IgE Western Blot

**Figure 6:**
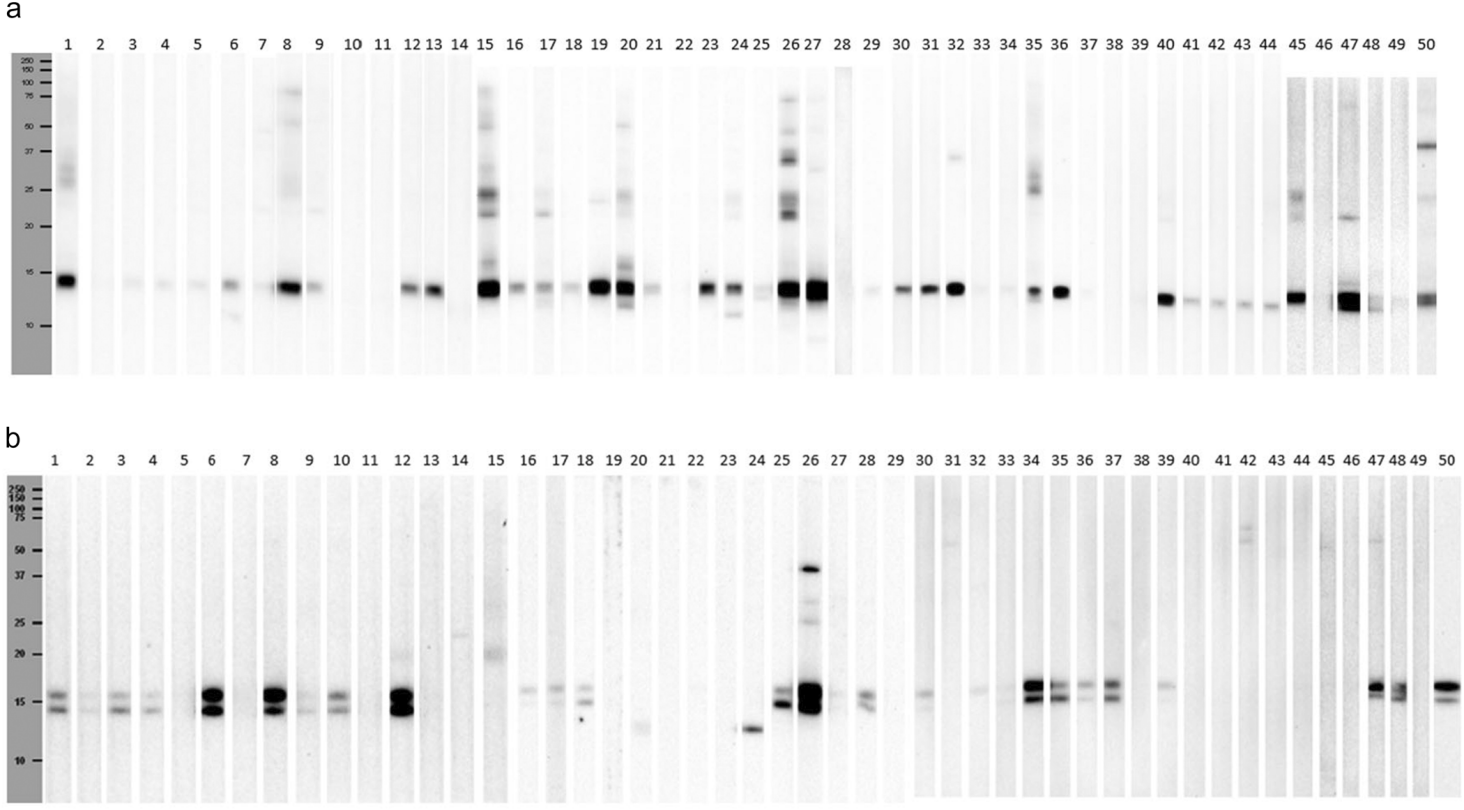
(a) Western blot *Dermatophagoides pteronyssinus*. (b) Western blot *Lepidoglyphus destructor*.

Western blot to Dpt and Ldt of selected patients showed different patterns of sensitization (Figure 6a,b). A marked stain intensity was found at 14–15 kDa for Dpt in almost all subjects (> 70%), followed by several proteins recognized mainly at 24–25 kDa. Concerning Ldt, more than 50% of the patients displayed a notable couple of proteins recognized about 14–15 kDa.

## 3 Discussion

In our study, it has been observed that the most prevalent HDM allergens in our geographical area are Der p 1: 70% (slightly higher than in Coruña, 66%), Der p 2: 84% (higher than in Coruña 78%), and Der p 23: 72% (slightly smaller than in Coruña, 86%) standing out above all of them [3], which exceeds the prevalence of other parts of the world [13, 14]. In addition to the higher prevalence, they had the highest serodominance values [15]: 22.4, 30.2 and 14.9 kU_A_/L for Der p 1, 2, and 23, respectively. We also found high prevalences of Lep d 2 (56%) and Tyr p 2 (54%).

Considering the 19 allergens of the different species included in the panel, 3 patients (6%) did not recognize any. Only 1 patient (2%) was monosensitive, recognizing Der p 23; 2 patients recognized 2 allergens; 2 patients 3; 3 patients 4; 4 patients 5; 6 patients 6; 6 patients 7; 4 patients 8; 5 patients 9; 5 patients 10; 3 patients 11; 2 patients 12; 2 patients 13 and 1 patient 14 allergens.

More than half of the patients (60%) recognized Der p 1, 2, and 23, alone or their combinations at the same time, 90% of the patients recognized any of the 3. Only 10% of the patients didn't recognize any of the 3.

On the other hand, if we analyse the combinations of the 9 molecules of Dpt (1,2,23,5,7,10,11,20,21), 26% of the patients presented sensitization to 3 molecules of Dpt, with Der p 1 + 2 + 23 being the main combination. In second and third place are the patients who presented sensitization to 5 molecules (20%) and those who presented sensitization to 6 molecules (18%). It should be noted that in patients sensitized to 3 molecules, the main combinations are between Der p 1 + Der p 2 together with a third molecule. All patients sensitized to 5 molecules present the combination Der p 1 + 2 + 23 together with two other molecules. The main combination presented by patients is Der p 1 + 2 + 21 + 23 + 5 + 7 (6 molecules), which represents 16% of patients.

The prevalence of the different Dpt molecules analysed is similar in the group of patients with intermittent and persistent AR, without significant differences. Regarding the severity of RA, the prevalence of Der p 1 is similar in mild or moderate patients. However, the prevalence of Der p 2 and Der p 23 is higher in patients with severe RA, while Villalta found a greater association of Der p 23 with rhinitis [12].

No statistical differences were found in the HDM sensitization values among asthmatics. In our study, a positive Der p 23 is not a risk factor to be asthmatic, contrarily to the Italian study [16].

In contrary to what described by López‐Rodríguez [3], all patients with asthma had sensitivity to Der p 2, according to what described by Weghofer [17] and Villalta [12] and more than 80% to Der p 1 and Der p 23. It should be noted that in the group of patients without asthma, sensitivity to Der p23 is considerably reduced by up to 50%. This reference is very close to statistical significance.

We found percentages of positivity like those described in the literature for Der p 5 (44%), Der p 7 (34%), Der p 21 (40%) [12, 18]. Levels of Der p 20 (4%), Der p 10 (4%) and Der p 11 (0%) were very small and like previous studies made in Galicia [3].

In our patients, we found specific IgE against group 1 and/or group 2 allergens of both Dpt and Dfar, similar to the data obtained by Barber et al. [18]. Identical for group 2 (84%) and very similar for group 1, 70% and 56% respectively for Dpt and Dfar.

In conclusion, the evaluation of specific IgE results in a comprehensive panel of allergens allows differentiation of various serological reactivity profiles with their clinical expression and improves patient management.

In the population studied, most patients (90%) recognize Der p 1, 2, and 23, alone or in any of their combinations, with very high sIgE values, 22.4; 30.2 and 14.9 kU_A_/L respectively. The high prevalence of sensitization to Der p 23 (72%) stands out, that all asthmatic patients are sensitive to Der p 2 and that sensitization to Der p 23 in non‐asthmatic patients drops to 50%.

The patient sample collected in Lugo was predominantly female (64%) and had a mean age of 28.5 years (SD 14.8). Most were adults (74%). In addition, the statistically significant differences observed in a few parameters (higher prevalence of Der p 2 compared to Der p 1 in moderate allergic rhinitis and absence of Der p 1, 2, and 23 in mild compared to moderate allergic rhinitis) should be studied in bigger populations, where a bigger number of statistically significant differences may be observed.

Considering that Galicia includes several well‐differentiated climatic zones, it would be advisable to carry out a study that either includes all the regions of the province or groups them by their climatic conditions.

## Author Contributions

**Luis Alfredo González:** conceptualization, data curation, investigation, methodology. **Ramón Núñez:** conceptualization, data curation, investigation, methodology. **Raquel Lopez:** conceptualization, data curation, investigation, methodology. **Joaquín Martín:** conceptualization, data curation, investigation, methodology. **Nicola Giangrande:** conceptualization, data curation, investigation, methodology. **Antonio García‐Dumpierrez:** conceptualization, data curation, investigation, methodology. **David Rodríguez:** formal analysis, supervision, validation, writing – original draft. **Ricardo Palacios:** conceptualization, validation, writing – original draft, writing – review and editing.

## Ethics Statement

The study was conducted in accordance with the Declaration of Helsinki and approved by the Institutional Ethics Committee Complejo Hospitalario A Coruña on March 25, 2021 (code 2021/169).

## Consent

All subjects involved in the study gave informed consent, and the patients gave written informed consent to publish this paper.

## Conflicts of Interest

The authors declare no conflicts of interest.

## Data Availability

The data that support the findings of this study are available from the corresponding author upon reasonable request.
